# Clinical Presentation, Tumor Characteristics, and Management of Intradiverticular Transitional Cell Carcinoma of the Urinary Bladder: A Systematic Review

**DOI:** 10.7759/cureus.62974

**Published:** 2024-06-23

**Authors:** Parisa Aijaz, Kulsoom Farooqi Baloch, Haseeb Faiz, Abdul Karim Durvesh, Syeda Javeria Tirmizi, Maimoona Khan, Hassan Sohail, Saad Khalid, Muhammad A Niazi, Amir Kamran

**Affiliations:** 1 Internal Medicine, Charleston Area Medical Center, Charleston, USA; 2 Internal Medicine, Hofstra University, Hempstead, USA; 3 Internal Medicine, Allama Iqbal Medical College, Lahore, PAK; 4 Internal Medicine, Dow University of Health Sciences, Karachi, PAK; 5 Hematology and Oncology, Charleston Area Medical Center, Charleston, USA

**Keywords:** transitional cell carcinoma, oncology, urology, a systematic review, urinary bladder diverticulum

## Abstract

Intradiverticular transitional cell carcinoma (TCC) of the bladder poses unique challenges due to its presentation within the bladder diverticula. This review synthesizes current knowledge on the diagnosis and management of this condition, emphasizing the need for early detection to optimize patient outcomes. The literature underscores the importance of tailored treatment strategies, ranging from radical surgeries to adjuvant chemotherapy, to combat the aggressive nature of intradiverticular TCC. Additionally, stringent post-treatment surveillance protocols are vital in addressing high recurrence rates. Future research directions include biomarker identification, comparative efficacy studies of treatment modalities, and the exploration of innovative therapeutic approaches such as immunotherapy. Longitudinal studies analyzing patient outcomes will provide valuable insights into survival rates and quality of life post-treatment, informing future clinical guidelines. This comprehensive review aims to enhance understanding and management strategies for intradiverticular TCC, paving the way for improved patient care and outcomes in this challenging form of bladder cancer.

## Introduction and background

A bladder diverticulum is an outpouching of the bladder wall that forms as a result of mucosal herniation into the muscular layer of the bladder wall [1,2]. Urine stasis in the diverticulum due to decreased contractility and chronic irritation can lead to inflammation and increase neoplasm risk, exacerbated by exposure to carcinogens like aromatic amines [3]. TCC, the most common bladder cancer subtype, primarily arises from the urothelium. Intradiverticular TCC, located within the diverticula, presents unique clinical challenges necessitating specialized diagnostic and therapeutic approaches. Understanding TCC's epidemiology, clinical aspects, and optimal management is crucial [2]. Despite its rarity, the aggressive nature and challenging location of intradiverticular TCC require careful monitoring. Given the lack of standardized treatment criteria, a systematic review is essential to consolidate data, identify gaps, and provide evidence-based recommendations for clinical decision-making. This review aims to compile literature on intradiverticular TCC, focusing on frequency, clinical characteristics, diagnostic precision, and treatment options. By conducting a thorough literature analysis, we intend to clarify optimal diagnostic methods and key prognostic factors and assess the effectiveness of both non-surgical and surgical treatment options.

## Review

Methods

We performed a systematic literature review of the databases, MEDLINE/PubMed, Scopus, Google Scholar, clinicaltrials.gov, and Cochrane CENTRAL by the Preferred Reporting Items for Systematic Review and Meta-Analysis Statement (PRISMA 2020) [4].

Search strategy

The search strategy was developed using the following keywords and corresponding MeSH terms without applying any filters (transitional cell carcinoma OR transitional cell carcinoma of bladder OR transitional cell cancer OR bladder carcinoma OR urothelial cancer OR bladder cancer OR urinary bladder cancer OR bladder carcinoma OR bladder tumor OR bladder neoplasm) AND (intradiverticular OR diverticulum) AND (management). We then combined and deduplicated the search results.

Eligibility criteria

Inclusion criteria included studies with interdiverticular bladder carcinoma that included management details, studies that fulfilled the database criteria, and studies that were in the English language. Excluded studies included those with non-English text, nonhuman studies, unavailable full texts, and carcinoma outside the bladder diverticula.

Study selection and screening

The studies with patients confirmed to have bladder diverticulum resulting in bladder carcinoma were included and the efficacy of their treatment plans was assessed. The titles and abstracts were screened by two reviewers (Tirmizi J and Khan M), and the articles were assessed for validity. The studies were then selected for inclusion and exclusion by senior author and consensus was reached by discussion. Any discrepancies were resolved by consultation with a third reviewer (Khalid S). 

Data extraction and quality assessment

A database with patient characteristics was made, which included the number of patients, age, sex, presenting complaints, history of smoking, occupational risk factor, urine DR, urine cytology, tumor characteristics, histological characteristics, treatment, and outcome. Studies were assessed for risk of bias using the Newcastle Ottawa scale (NOS). Selection, ascertainment, causality, and reporting of case reports were assessed using this tool, and the studies were classified as low risk, medium risk, and high risk as shown in the table.

Data analysis

Statistical analysis was then performed to assess the best treatment outcome in the case of interdiverticular bladder carcinoma using means to report continuous variables and percentages and frequencies for dichotomous variables. Meanwhile, similarities in the tumor characteristics and treatment plans between the cases were also described narratively.

Quality assessment

Using Murad et al.’s standardized technique for assessing case reports and case series quality, four (7.4%) of the studies had a high risk of bias, 13 (24.07%) had a medium risk, and 38 (68.5%) had a low probability of bias [5]. A total of seven studies (12.9%) were unable to offer sufficient support for our desired outcomes. Even nevertheless, 40 (74.07%) of the studies offered sufficient and unambiguous information on the exposure. Twenty-one studies (33.8%) did not follow-up with enough patients to determine the clinical outcome in full. Complete quality assessment for every item can be found in the table below.

Risk assessment of case studies

Case studies are inherently biased. Standardized instruments, on the other hand, have been developed to evaluate the methodological quality of case report systematic reviews. We employed the modified NOS developed by Murad et al. to rate the quality of the case series and reports that were part of the investigation [5]. This instrument evaluates four domains, selection, ascertainment, causation, and reporting, through a set of eight questions. The test's questions four, five, and six were excluded since they have little bearing on our subject and are primarily relevant to instances of adverse drug responses as the tool describes them [4]. The following five questions determined the cumulative score, which determined the level of bias risk for each article: "high risk," "medium risk," or "low risk." We regarded case reports and case series as having a low risk of bias if they received a score of four or five on the quality evaluation questions. A score of three indicated a medium risk of bias for an article, and a score of less than three indicated a high risk of bias (Tables 1, 2).

**Table 1:** Risk assessment of case studies

Study	Does the patient(s) represent(s) the whole experience of the investigator (center) or is the selection method unclear to the extent that other patients with similar presentation may not have been reported?	Was the exposure adequately ascertained d?	Was the outcome adequately ascertained?	Was follow-up long enough for outcomes to occur?	Is the case(s) described with sufficient details to allow other investigators to replicate the research or to allow practitioners make inferences related to their own practice?	Risk of bias
Abiad [6]	Yes	No	Yes	Yes	No	Medium risk
Abdul Rahman [7]	No	Yes	No	No	Yes	High risk
Agarwal [8]	Yes	Yes	Yes	Yes	Yes	Low risk
Al Hajjaj [9]	Yes	Yes	Yes	Yes	Yes	Low risk
Baniel [10]	No	Yes	Yes	Yes	Yes	Low risk
Andro [11]	Yes	Yes	Yes	Yes	Yes	Low risk
Bjerklund [12]	Yes	Yes	Yes	Yes	Yes	Low risk
Bourgi [13]	No	No	Yes	Yes	No	High risk
Cramer [14]	Yes	Yes	Yes	No	Yes	Low risk
Dong [15]	Yes	No	Yes	Yes	Yes	Low risk
Dragsted [16]	Yes	Yes	No	No	Yes	Medium risk
Durfee [17]	Yes	Yes	No	No	Yes	Medium risk
Elands [18]	Yes	Yes	Yes	Yes	Yes	Low risk
Evans [19]	Yes	Yes	No	No	No	Medium risk
Faysal [20]	Yes	No	Yes	Yes	No	Medium risk
Fu [21]	No	Yes	Yes	No	No	High risk
Garcia [22]	Yes	Yes	No	No	Yes	Medium risk
Garzoto [23]	No	No	Yes	Yes	No	Medium risk
Grubišić [24]	Yes	Yes	Yes	Yes	Yes	Low risk
Haecker [25]	Yes	Yes	Yes	Yes	Yes	Low risk
Holck [26]	Yes	Yes	Yes	Yes	Yes	Low risk
Knappenberger [27]	Yes	Yes	Yes	Yes	Yes	Low risk
Kong [28]	Yes	Yes	Yes	Yes	No	Low risk
Kelalis [29]	Yes	Yes	Yes	Yes	No	Low risk
Knezevic [30]	No	Yes	Yes	Yes	No	Medium risk
Madison [31]	Yes	Yes	Yes	No	Yes	Low risk
Matta [32]	Yes	Yes	No	No	Yes	Medium risk
Mayer [33]	Yes	Yes	Yes	Yes	Yes	Low risk
Md Noh [34]	Yes	Yes	Yes	Yes	Yes	Low risk
Melekos [35]	Yes	Yes	Yes	yes	Yes	Low risk
Mittal [36]	Yes	Yes	Yes	No	Yes	Low risk
Moinzadeh [37]	Yes	Yes	Yes	No	No	Medium risk
Montague [38]	Yes	No	Yes	Yes	Yes	Low risk
Moussa [39]	Yes	Yes	Yes	No	Yes	Low risk
Nanbu [40]	Yes	Yes	Yes	No	No	Medium risk
Omeroglu [41]	Yes	No	Yes	Yes	Yes	Low risk
Ozguven [42]	Yes	No	Yes	No	Yes	Medium risk
Pearlman [43]	Yes	Yes	Yes	Yes	Yes	Low risk
Rabin [44]	Yes	Yes	Yes	Yes	Yes	Low risk
Raheem [45]	Yes	Yes	Yes	Yes	Yes	Low risk
Redman [46]	Yes	Yes	Yes	No	Yes	Low risk
Kumar Sah [47]	Yes	Yes	Yes	No	Yes	Low risk
Siegel [48]	Yes	No	Yes	No	Yes	Medium risk
Shah [49]	Yes	Yes	Yes	Yes	Yes	Low risk
Shigehara [50]	Yes	Yes	Yes	No	Yes	Low risk
Escandon [51]	Yes	No	Yes	Yes	Yes	Low risk
Thwaini [52]	Yes	Yes	Yes	No	Yes	Low risk
Tonzi [53]	Yes	No	Yes	Yes	yes	Low risk
Tsuboi [54]	Yes	Yes	Yes	No	Yes	Low risk
Tudor [55]	Yes	No	No	No	Yes	High Risk
Wang [56]	Yes	Yes	Yes	Yes	Yes	Low risk
Lembo [57]	Yes	Yes	Yes	Yes	Yes	Low risk
Labanaris [58]	Yes	No	Yes	Yes	Yes	Low risk
Muellner [59]	Yes	Yes	Yes	Yes	Yes	Low risk

**Table 2:** Risk assessment of cohort studies ^-No^ ^*Yes^

Study	Representativeness of exposed cohort	Selection of non-exposed cohort	Ascertainment of Exposure	Demonstration that outcome of interest was not present at the start of study	Comparability of cohorts on the basis of design or analysis	Assessment of outcome	Was follow-up long enough for outcome to occur	Adequacy of follow-up	Risk of bias
Golijanin [60]	-	-	*	*	-	*	*	*	Medium Risk
Hu [61]	-	-	*	*	-	-	*	*	High risk
Di Paolo [62]	-	*	-	*	-	*	*	*	Medium risk
Voskuilen [63]	*	*	*	*	*	*	-	*	Low risk
Zhong [64]	*	*	*	*	*	*	*	*	Low risk
Targett [65]	*	*	*	-	*	*	*	*	Low risk
Yu [66]	*	*	-	*	*	*	*	*	Low risk

Screening

**Figure 1:**
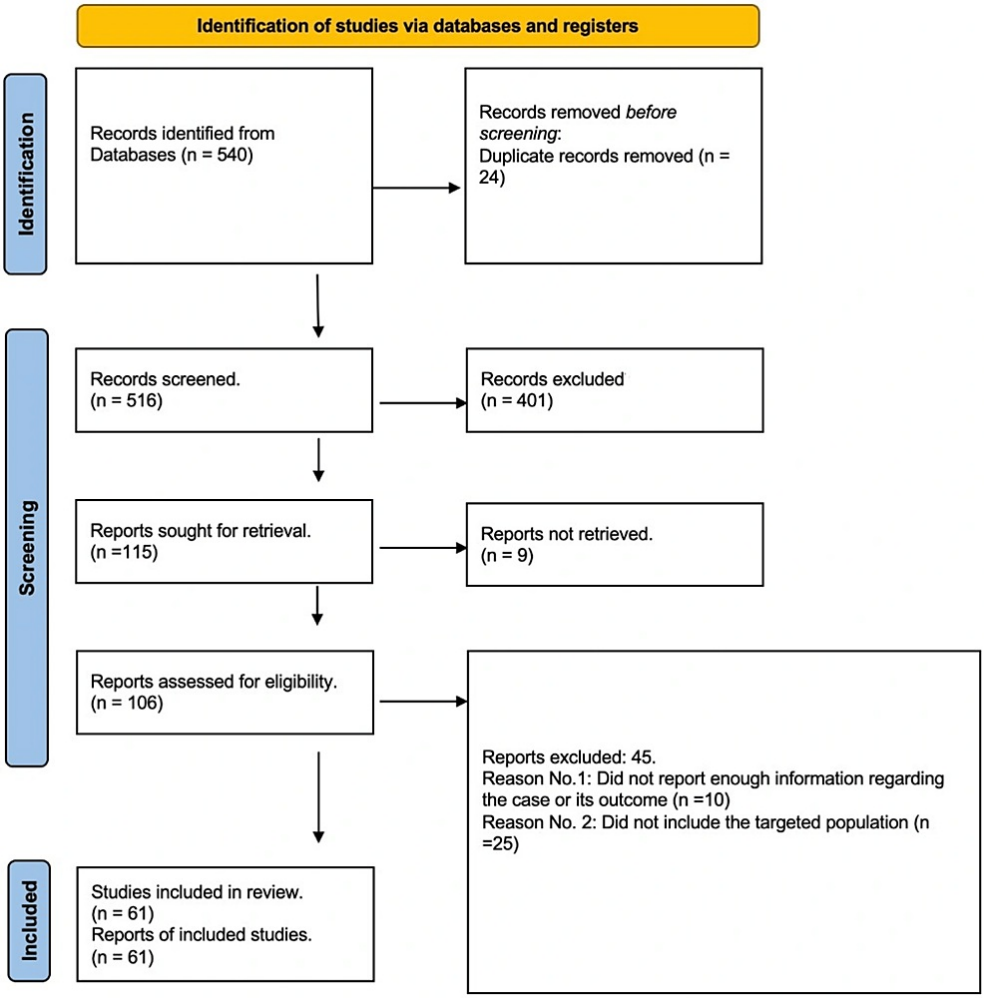
Screening process

The total number of articles identified was 540 articles in Scopus, Google Scholar, Science Direct, and PubMed. These articles were identified using keywords (transitional cell carcinoma OR transitional cell carcinoma of bladder OR transitional cell cancer OR bladder carcinoma OR urothelial cancer OR bladder cancer OR urinary bladder cancer OR bladder carcinoma OR bladder tumor OR bladder neoplasm) AND (intradiverticular OR diverticulum) AND (management). Before the screening process, 24 duplicate articles were removed. After removing duplicates, 516 articles were screened using title and abstract, and 401 articles were screened out. Nine articles were removed because of the unavailability of the full text. In total, 106 articles were deemed eligible for the final screening and data extraction (Figure 1).

Results

A total of 3361 patients were included in this systematic review, 2920 (86.8%) of which were men, and 441 (13.2%) patients were female. The mean age was 64.21±8.2 years old. Clinical features (Table 3) were reported in N=271 patients. Most common clinical symptoms included hematuria (N=208, 76.7%), including gross hematuria (N=108, 52%), microscopic hematuria (N=2, 0.7%), unspecified hematuria (N=98, 47%), and urinary tract symptoms (N=63, 23.2%), including urgency (N=9), frequency (N=10), prostatitis (N=12), and urinary tract obstruction (N=13).

**Table 3:** Reported clinical features

Reported clinical features	(N=271) n (%)
Hematuria (unspecified)	98 (47%)
Gross hematuria	108 (52%)
Microscopic hematuria	2 (0.7%)
Urinary urgency	9 (3.3%)
Urinary frequency	10 (3.6%)
Dysuria	5 (1.8%)
Urinary tract infection	7 (2.5%)
Urinary tract obstruction	13 (4.8%)
Lower abdominal and pelvic pain	5 (1.8%)
Prostatitis	12 (4.4%)
Priapism	1 (0.3%)
Weight loss	1 (0.3%)

Tumor characteristics

Type of carcinoma was defined in most patients (N=2366), out of which, the most common type of carcinoma identified was urothelial carcinoma (2274, 96.1%). Other major carcinomas identified were squamous cell carcinoma in 34 patients, papillary cell carcinoma in 21 patients, and adenocarcinoma in 10 patients (Table 4).

**Table 4:** Type of carcinoma

Type of carcinoma	(N=366) n (%)
Urothelial carcinoma	2274 (96.1%)
Squamous cell carcinoma	34
Papillary cell carcinoma	21
Adenocarcinoma	10
Sarcomatoid carcinoma	9
Invasive cell carcinoma	3
Carcinoma in situ	3
Neuroendocrine carcinoma	1
Sarcoma	1
Small cell carcinoma	1
Leiomyosarcoma	1
Verrucous carcinoma	1
Undifferentiated carcinoma	1
Osteosarcoma	1
Oat cell carcinoma	1
Mixed cell carcinoma	1
Carcinosarcoma	2

Stage of tumor

A total of 2252 patients in 61 studies had their stages defined (Table 5). Most of the patients (N=1198) presented with stage T2 disease. The remaining patients had stage T1 (N=647), Ta (N=200), T4 (N=117), and T3 (90).

**Table 5:** Stage of the tumor

Tumor stage	(N=2252) n (%)
Ta	200 (8.8%)
T1	647 (287%)
T2	1198 (53.2%)
T3	90 (4.0%)
T4	117 (5.2%)

Grade of tumor

Only 294 patients in the included studies had their grading defined (Table 6).

**Table 6:** Grade of the tumor

Tumor grade	(N=287) n (%)
Grade 1	60 (20.9%)
Grade 2	40 (13.9%)
Grade 3	172 (59.9%)
Grade 4	15 (5.2%)

Gross tumor appearance

Among the studies that reported the gross appearance of the tumors, most patients had a polyploid shape (N=21) followed by a papillary growth tumor (N=14). The mean size of the tumor in the maximum dimension was 45 mm and the mean size of the diverticulum in the maximum dimension was 60.5 mm (Table 7).

**Table 7:** Gross appearance of tumors

Gross appearance of tumor	(N=59), n (%)
Necrotic	3 (5%)
Nodular	1 (1%)
Papillary growth	14 (23%)
Pedunculated	4 (6%)
Sessile	12 (20%)
Polyploid	21 (35%)
Solid globular mass	3 (5%)
Undifferentiated	1 (1%)

Cystoscopy findings

Cystoscopy findings were reported for 71 patients. Gross tumor was reported in 56% of cystoscopies. A diverticulum was reported above the right ureteric orifice in 8.4% of patients. Further cystoscopy findings are detailed (Table 8).

**Table 8:** Cystoscopy findings

Cystoscopy findings	(N=71) n (%)
Normal bladder mucosa	2 (2.8%)
Cystoscopy was not diagnostic	2 (2.8%)
External compression felt during cystoscopy	5 (7.0%)
Enlarged prostate	3 (4.2%)
Blood clots in the bladder or diverticulum	4 (5.6%)
Diverticulum above right ureteric orifice	6 (8.4%)
Diverticulum above left ureteric orifice	3 (4.2%)
Suspected tumor in the diverticulum	40 (56%)
Suspected tumor was in the bladder wall	3 (4.2%)
Displaced bladder	2 (2.8%)
Double collecting system	1 (1.4%)

Biopsy findings

The biopsy results of the mass showed varied findings, ranging from cellular atypia with prominent mitotic figures to locally advanced squamous cell carcinoma (Table 9). In some cases, the biopsy was inconclusive or did not yield positive results. 

**Table 9:** Biopsy findings TCC, transitional cell carcinoma

Descriptive biopsy findings
Atypical hyperchromatic transitional epithelial cells with areas of necrosis
Atypical, oval to spindle-shaped cells with prominent mitotic activity in central parts of the tumor; the osteoid was mineralized and deposited as irregular trabeculae with malignant osteocytes within lacunae
Biopsy was not successful
Cellular atypia with prominent mitotic activity and disrupted stratification
Hypercellular with hyperchromatic nuclei with resemblance to oat cells
Inflammatory and necrotic cells with occasional spindle-shaped cells in between
Infiltrating lamina propria
Locally advanced squamous cell carcinoma
Necrotic TCC
Pink-tan to hemorrhagic soft and rubbery tissue
Sheets and nests of small neuroendocrine cells with dense fibrous stroma. Positive for synaptophysin
Small round cells in the form of uniform sheets and nest
Tan-brown mucosa with some gray-white lesions, squamous features, signet ring cells
Trophoblastic giant cells
Undifferentiated spindle-shaped and pleomorphic stromal cells

Histological features

The histological features included polyploid (40%), papillary growth (35%), and mixed patterns (7%). Muscle invasion was reported in 41% of the patients, and carcinoma in situ (CIS) was reported in 31% of patients. The various histological findings (Table 10) and immunohistochemical staining (Table 11) are detailed.

**Table 10:** Histologic features CIS, carcinoma in situ

Histological features of the tumors	(N=261) n (%)
Muscle invasion	109 (41%)
Lymphovascular invasion	5 (1.7%)
CIS	65 (24%)
Noninvasive	82 (31%)
Histology pattern of the biopsy	(N=42) n (%)
Diffuse	1 (2.8%)
Exophytic	1 (2.8%)
Necrotic	1 (2.8%)
Hyperplastic cells	1 (2.8%)
Mitotic activity	1 (2.8%)
Mixed pattern	3 (7%)
Papillary pattern	15 (35%)
Poorly differentiated	1 (2.8%)
Polyploid1	17 (40%)
Tublocystic	1 (2.8%)

**Table 11:** Immunohistochemical staining EMA, epithelial membrane antigen; CEA, carcinoembryonic antigen; SMA, smooth muscle actin; PSA, prostate-specific antigen

Immunohistochemical stains	
Cytokeratin 5	2/2 (100%)
Cytokeratin 6	2/2 (100%)
Cytokeratin 7	1/2 (50%)
Cytokeratin 20	3/4 (75%)
Cytokeratin AE1/ AE3	3/3 (100%)
Cytokeratin D	1/1 (100%)
p63	6/6 (100%)
p53	1/1 (100%)
EMA	2/2 (100%)
CEA	1/2 (50%)
SMA	3/3 (100%)
S-100 protein	1/1 (100%)
Pankeratin	1/1 (100%)
Vimentin	4/5 (80%)
Synaptophysin	8/8 (100%)
PSA	0/2 (0%)
Ki-67	1/1 (100%)
Chromogranin A	5/6 (83%)
CD 56	1/1 (100%)
Desmin	1/1 (100%)
Prostatic acid phosphatase	0/1 (0%)
Leukocyte common antigen	0/1 (0%)
Intracytoplasmic mucin	0/1 (0%)
Anaplastic lymphoma kinase	0/1 (0%)

Treatment

The patients underwent a variety of treatments including surgical and nonsurgical interventions (Tables 12, 13). Non-surgical interventions included chemotherapy, radiotherapy, or a combination of both. Surgical intervention included radical cystectomy (28.4%), diverticulectomy 14%), and transurethral resection (23.9%). Among the patients who received chemotherapy, 92% received adjuvant systemic chemotherapy. 70% of those who received intravesical therapies, got Bacillus Calmette-Guerin (BCG).

**Table 12:** Surgical intervention

Surgical intervention	(N=489) n (%)
Patient refused surgery	2 (0.4%)
Chemoradiotherapy	3 (0.6%)
Diverticulectomy	70 (14%)
Radical cystectomy	139 (28.4%)
Total cystectomy	5 (1%)
Partial cystectomy	106 (21%)
Transurethral resection	117 (23.9%)
Tumor resection through cystostomy	14 (2.8%)
Tumor resection through laparotomy	1 (0.2%)
Prostatectomy	3 (0.6%)
Cyst prostatectomy	2 (0.4%)
Radiotherapy alone	5 (1%)
Lymphadenectomy performed along with other procedures	22 (4.4%)

**Table 13:** Chemotherapy regimen BCG, Bacillus Calmette-Guerin

Systemic chemotherapy	(N=52) n (%)
Adjuvant	48 (92%)
Neo Adjuvant	3 (5.7%)
Refractory to chemotherapy	1 (1.9%)
Intravesical therapy	(N=33) n (%)
Patients who received BCG	23 (70%)
Patients who received other agents	10 (30%)

Outcomes

The outcome of the studies was reported as alive or dead at the last follow-up period. The mean follow-up period was 22.3 months, the range was 1-110 months, and the median follow-up period was 16 months. 52% of the patients were alive at the last follow-up period and 45% of patients died at the last follow-up period. The rest were lost to the follow-up. Most of the patients died due to the original disease they had (42% out of 45%). Among the patients who were alive, 37% out of 52% were alive and disease-free. Around 83 patients had a recurrence of the disease and out of these, 73.4% had local recurrence while the rest of the patients developed metastatic disease (Table 14).

**Table 14:** Study outcomes

Study outcome	(N=352) n (%)
Total patients alive at the last follow-up period	180 (52%)
Patients alive without disease	129 (37%)
Patients alive with disease	49 (14%)
Total patients dead at the last follow-up period	157 (45%)
Patients died due to original disease	145 (42%)
Patients died due to recurrence of disease	5 (2.4%)
Patients died due to some other disease	7 (2%)
Patients lost to follow-up period	8 (2.3%)

Discussion

The management of intradiverticular TCC of the bladder presents a unique challenge in terms of management due to its location within the bladder diverticulum. The management of this condition requires a multidisciplinary approach involving urologists, oncologists, radiologists, and sometimes pathologists. One of the primary considerations in managing intradiverticular TCC is the anatomical complexity and potential complications associated with the diverticulum itself. The review encompassed a comprehensive analysis of 3361 patients, with a notable predominance of male subjects, constituting 86.8% of the cohort. The mean age of the patients was 64.21 years, indicative of an older population affected by this condition. Findings from this systematic review indicate that the clinical symptoms of intradiverticular TCC of the bladder vary widely and are frequently ambiguous. Hematuria was the most common symptom, affecting more than three-quarters of the patients with clinical features, followed by irritative urinary symptoms, which were present in less than 10%. Other symptoms, such as urinary tract infection, urinary tract obstruction, lower abdominal and pelvic pain, prostatitis, priapism, and weight loss, were rare and occurred in less than 5% of the patients. These findings suggest that intradiverticular TCC of the bladder has a heterogeneous clinical spectrum and may be easily overlooked or misdiagnosed, especially in patients with mild or asymptomatic disease. The high prevalence of hematuria among patients with intradiverticular TCC of the bladder indicates that this symptom should prompt further investigation for the presence of bladder diverticula and TCC. However, the diagnostic value of hematuria may be limited by the lack of specificity and the variation in the type and severity of bleeding [67]. The low frequency of irritative urinary symptoms, urinary tract infection, and obstruction suggests that these features are not reliable indicators of intradiverticular TCC of the bladder. Moreover, these symptoms may be confounded by other benign or malignant conditions affecting the lower urinary tract, such as benign prostatic hyperplasia, prostate cancer, bladder stones, or urothelial carcinoma [68]. Therefore, these features should not be used to rule out the possibility of intradiverticular TCC of the bladder in patients with hematuria or other risk factors. The rare occurrence of pain, prostatitis, priapism, and weight loss among patients with intradiverticular TCC of the bladder implies that these features are either late manifestations of the disease or unrelated comorbidities. These features may reflect the invasion of the tumor into adjacent structures, such as the prostate, seminal vesicles, urethra, or pelvic organs, or the presence of distant metastases, such as the lymph nodes, bones, or lungs [69]. Therefore, these features should alert the clinician to the possibility of advanced or aggressive disease and prompt further staging and treatment.

Urothelial carcinoma was identified as the predominant type, making up 96.1% of the cases reviewed. This finding is consistent with the established understanding that urothelial carcinoma is the most common form of bladder cancer, known for its potential for recurrence and progression, necessitating vigilant monitoring and management strategies [67]. In addition to urothelial carcinoma, the review identified several other carcinoma types, though in significantly smaller numbers. These include squamous cell carcinoma, papillary cell carcinoma, and adenocarcinoma, among others. The presence of squamous cell carcinoma and adenocarcinoma, while less common, is notable and aligns with literature indicating their association with specific risk factors and potentially different clinical courses compared to urothelial carcinoma [70]. Papillary cell carcinoma, often considered a subset of urothelial carcinoma, is recognized for its distinct growth patterns and potentially more favorable prognosis when identified early [71]. The identification of rare carcinoma types such as sarcomatoid carcinoma, neuroendocrine carcinoma, and carcinosarcoma, although in minimal numbers, underscores the histological diversity within bladder cancers and highlights the complexity of managing this disease. Sarcomatoid carcinoma, for instance, is known for its aggressive behavior and poor prognosis, emphasizing the need for aggressive treatment strategies [72]. Similarly, the rare occurrence of neuroendocrine carcinoma and carcinosarcoma in bladder cancer patients points to the necessity for specialized diagnostic and therapeutic approaches to manage these aggressive cancer subtypes effectively.

The findings also revealed that a substantial number of patients, 53.2%, were diagnosed with stage T2 disease, indicating a tendency for intradiverticular TCC to be diagnosed at a relatively advanced stage. This stage is characterized by muscle invasion, which is a critical threshold for treatment decision-making, often necessitating more aggressive treatment options like radical cystectomy or systemic chemotherapy [67]. The distribution of other stages, with T1 (28.7%) and Ta (8.8%) being less common, underscores the invasive nature of intradiverticular TCC, contrasting with the generally more favorable prognosis associated with non-muscle invasive bladder cancer (NMIBC). The grading of tumors, reported in 294 patients, showed a predominance of high-grade (Grade 3) tumors (59.9%), followed by Grade 1 (20.9%), Grade 2 (13.9%), and Grade 4 (5.2%) tumors. This distribution highlights the aggressive behavior of intradiverticular TCC, with a significant proportion of tumors being high-grade, which is associated with a higher risk of progression and poorer outcomes [73]. Gross appearance data from the studies indicated a variety of tumor morphologies, with polyploid shapes (35%) and papillary growth (23%) being the most common. The mean size of the tumors was 45 mm, and the mean size of the diverticula was 60.5 mm, suggesting that intradiverticular TCC tends to present as sizable masses within relatively large diverticula. This finding is crucial for surgical planning and may influence the choice of surgical approach and the extent of resection required [74]. Cystoscopy findings further complemented these observations, with a gross tumor visible upon entering the bladder in 56% of cases, indicating that cystoscopy is a valuable diagnostic tool for identifying intradiverticular TCC. The presence of diverticula above the right ureteric orifice in 8.4% of patients and external compression during cystoscopy in 7% of cases highlight the anatomical challenges and potential complications associated with this condition, such as ureteral obstruction or involvement of surrounding structures [75].

The biopsy findings demonstrate a broad spectrum of cellular abnormalities, ranging from atypical hyperchromatic transitional epithelial cells with areas of necrosis to locally advanced squamous cell carcinoma. Some biopsies were unsuccessful or did not yield definitive results, indicating the challenges in obtaining diagnostic tissue from these tumors. The diversity in biopsy findings, including the presence of osteoid with malignant osteocytes, cellular atypia with prominent mitotic activity, and various forms of carcinoma, underscores the complex nature of intradiverticular TCC and its potential for diverse differentiation pathways. Histologically, the tumors exhibited a variety of patterns, with 40% showing a polyploid pattern, 35% papillary growth, and a notable proportion (41%) demonstrating muscle invasion. The presence of CIS in 31% of patients further highlights the aggressive potential of these tumors. The variety in histological patterns and the significant rate of muscle invasion and CIS further underscore the aggressive behavior of intradiverticular TCC, necessitating careful management strategies to address both local control and the risk of progression [67,76]. Immunohistochemical staining revealed a wide range of cell markers, with high expression levels of cytokeratins (CK5, CK6, AE1/AE3), p63, and synaptophysin, among others. This marker profile suggests that these tumors arise from epithelial cells, with synaptophysin indicating some degree of neuroendocrine differentiation. The lack of prostate-specific antigen (PSA) and prostatic acid phosphatase markers confirms these tumors do not stem from prostate tissue, aligning with their identification within the bladder. The variability in marker expression highlights the heterogeneity of intradiverticular TCC and may provide insights into tumor origin, differentiation, and potential therapeutic targets [73].

Surgical intervention is often the cornerstone of treatment for intradiverticular TCC. However, the approach to surgery can vary depending on factors such as the size and location of the tumor within the diverticulum, the presence of concomitant bladder pathology, and the overall health status of the patient. The review, involving 489 patients, highlights a diverse range of treatment modalities employed to manage this condition. Radical cystectomy was the most common treatment choice (28.4%), followed by partial cystectomy (21%) and transurethral resection (23.9%). These surgical options reflect the aggressive approach often required for managing bladder cancer, especially when muscle invasion is present. Diverticulectomy, a more conservative surgical option, was chosen in 14% of cases, indicating its role in selected patients with localized disease. Chemotherapy plays a crucial role in the management of bladder TCC, with 92% of patients undergoing adjuvant chemotherapy. The use of various chemotherapy combinations, including Cisplatin with Methotrexate, Etoposide, Epirubicin, Gemcitabine plus Carboplatin, and others, underscores the tailored approach to chemotherapy based on patient-specific factors and tumor characteristics. Intravesical therapy, predominantly with BCG (70%), highlights its importance in NMIBC to reduce recurrence and progression rates, as supported by the literature [67]. The outcomes of the study, with a median follow-up period of 16 months, show that 52% of patients were alive at the last follow-up, with 37% alive and disease-free. This outcome suggests a significant proportion of patients achieve a favorable response to treatment, although 45% of patients died, with the majority succumbing to the original disease. The recurrence rate of 83 patients, with 73.4% experiencing local recurrence, emphasizes the challenging nature of intradiverticular TCC and the need for vigilant post-treatment surveillance to detect and manage recurrences early.

## Conclusions

Our review on managing intradiverticular TCC highlights the varied clinical presentation of this condition. Early diagnosis is crucial for improving outcomes. Customized treatment plans, including radical surgeries and adjuvant chemotherapy, are essential. Robust post-treatment surveillance is key to addressing high recurrence rates. Future research should focus on biomarker identification, treatment efficacy comparisons, and novel therapies like immunotherapy for intradiverticular TCC. Longitudinal studies will offer insights into survival rates and quality of life post-treatment, guiding future treatment strategies for improved patient care.
